# Biomarkers of Metabolic Adaptation to High Dietary Fats in a Mouse Model of Obesity Resistance

**DOI:** 10.3390/metabo14010069

**Published:** 2024-01-20

**Authors:** Fadia Milhem, Leah M. Hamilton, Emily Skates, Mickey Wilson, Suzanne D. Johanningsmeier, Slavko Komarnytsky

**Affiliations:** 1 Plants for Human Health Institute, North Carolina State University, 600 Laureate Way, Kannapolis, NC 28081, USA; fjmilhem@ncsu.edu (F.M.); eskates@ncsu.edu (E.S.); mlwilso8@ncsu.edu (M.W.); 2 Department of Food, Bioprocessing and Nutrition Sciences, North Carolina State University, 400 Dan Allen Drive, Raleigh, NC 27695, USA; lhamilton@vsu.edu; 3 Department of Nutrition, University of Petra, 317 Airport Road, Amman 11196, Jordan; 4 College of Agriculture, Virginia State University, 1 Hayden Drive, Petersburg, VA 23806, USA; 5 United States Department of Agriculture-Agricultural Research Service, Southeast Area, Food Science and Market Quality & Handling Research Unit, North Carolina State University, 322 Schaub Hall, Box 7624, Raleigh, NC 27695, USA; suzanne.johanningsmeier@usda.gov

**Keywords:** body composition, metabolic inflexibility, metabolic rate, dietary fats, skeletal muscle remodeling, glucose tolerance, microbiome

## Abstract

Obesity-resistant (non-responder, NR) phenotypes that exhibit reduced susceptibility to developing obesity despite being exposed to high dietary fat are crucial in exploring the metabolic responses that protect against obesity. Although several efforts have been made to study them in mice and humans, the individual protective mechanisms are poorly understood. In this exploratory study, we used a polygenic C57BL/6J mouse model of diet-induced obesity to show that NR mice developed healthier fat/lean body mass ratios (0.43 ± 0.05) versus the obesity-prone (super-responder, SR) phenotypes (0.69 ± 0.07, *p* < 0.0001) by upregulating gene expression networks that promote the accumulation of type 2a, fast-twitch, oxidative muscle tissues. This was achieved in part by a metabolic adaptation in the form of blood glucose sparing, thus aggravating glucose tolerance. Resistance to obesity in NR mice was associated with 4.9-fold upregulated mitoferrin 1 (*Slc25a37*), an essential mitochondrial iron importer. SR mice also showed fecal volatile metabolite signatures of enhanced short-chain fatty acid metabolism, including increases in detrimental methyl formate and ethyl propionate, and these effects were reversed in NR mice. Continued research into obesity-resistant phenotypes can offer valuable insights into the underlying mechanisms of obesity and metabolic health, potentially leading to more personalized and effective approaches for managing weight and related health issues.

## 1. Introduction

Modern conveniences, such as energy-dense foods and sedentary lifestyles, contribute significantly to weight gain and difficulty in weight management [1]. Genetic factors, cultural associations with certain types of foods, environmental pollutants, socioeconomic disparities, as well as stigmatization and discrimination against individuals struggling with their weight can further exacerbate obesity [2]. The abundance of conflicting information about diets, exercise regimens, and weight loss methods may add to ineffective or unsustainable approaches for those who struggle with excessive weight. Though it is commonly thought that altering physical activity levels could balance energy intake and create a negative energy state, the extent of these compensatory mechanisms is frequently overestimated. For instance, an adult who consumes a 290 kcal meal would need to walk approximately 90 min, or 5 km, to reach a theoretical energy equilibrium [3]. A multifaceted approach involving education about nutrition and lifestyle choices, policy changes to promote healthier food production systems, and enhancing access to affordable, nutritious foods is a clear priority [4]. Additionally, personalized approaches that consider individual differences in genetics, metabolism, and behavioral patterns cannot be overlooked [5].

Resistance to obesity associated with the overconsumption of calorie-dense foods is a very interesting phenomenon [6]. Although the body can restrain food intake below the levels of maximum consumption, the precise mechanism that modulates this process remains unclear [7]. If food intake cannot be restrained for a variety of reasons, the flow of nutrients between metabolically active tissues and the timely oxidation of the energy substrates must be controlled. A small but constant margin of error of 175 kcal energy intake per day may translate to 3 kg body weight gain or loss per year, highlighting a critical need to understand individual variation in nutrient absorption and metabolism [8]. Resistance to body weight gain despite excessive calorie intake has been observed both in rodents and humans, yet it is unclear how skeletal muscle [9] and adipose tissue [10] cooperate in this process. Free fatty acids sourced from the adipose tissue (fasted state) and circulating dietary triglycerides (fed state) are the principal lipid types used for oxidation. Because in healthy states, at least in rodents, dietary lipids are slow to absorb and the majority of them are partitioned into the gut, liver, and skeletal muscle but not adipose tissue in the first 24 h following food intake [11], it is logical to conclude that differences in the level of muscle tissue physiology and fat oxidation are the primary contributors to the inefficient handling of dietary fats and their subsequent partition into the adipose tissue. Indeed, resistance to obesity was observed to be associated in part with the activation of mitochondrial oxidative pathways [12]. 

This concept challenges the traditional notion that all obese individuals are at equal risk for metabolic complications, as discussed elsewhere [13], and predicts a subset of people with obesity that presents with a more favorable metabolic profile that allows one to accumulate less visceral and ectopic fat and build up resilience to the detrimental metabolic effects commonly associated with obesity. This, however, may come at the expense of disturbances in carbohydrate metabolism [14]. These effects can occur independently of body weight and are observed in both lean and obese individuals, emphasizing the importance of metabolic health beyond weight alone. 

Our previous work suggested that the obesity-resistant phenotype in C57BL/6J mice was linked to increased oxygen consumption during the light (sleep) cycle, increased physical activity during the dark (active) cycle, and increased heat production during both cycles, and this was achieved at the expense of decreased exercise capacity [15]. In this study, we further explored the differences between obesity-prone and obesity-resistant phenotypes, focusing on lean body mass and a set of physiological and mitochondrial adaptations that may be responsible for these effects. We also attempted to correlate these findings with changes in fecal volatile metabolite profiles focusing on short-chain fatty acids and their esters. 

## 2. Materials and Methods

### 2.1. Animals and Diets

Male, 4-week-old C57BL/6J mice were purchased from the Jackson Laboratory (Bar Harbor, ME, USA) and housed with four animals per cage under controlled temperature (24 ± 2 °C) and light (12 h light–dark cycle, lights on at 7:00 a.m.). Immediately upon arrival, animals were allowed to adapt to new conditions for 7 days and handling the animals was performed daily to reduce the stress of physical manipulation. Mice were then randomized into groups with ad libitum access to Research Diets (New Brunswick, NJ, USA), including a low-fat diet (LFD, D12450J, 10 kcal % fat, 3.85 kcal/g, n = 32) or high-fat diet (HFD, D12492, 60 kcal % fat, 5.24 kcal/g, n = 44) for 6 weeks. Obese animals were further randomized into normally obese (HFD), non-responders (NRs), super-responders (SRs), or super-responders switched back to the low-fat diet (SRs-LFD) for an additional 8 weeks of the respective diets [15]. All animal experiments were performed according to procedures approved by the NC Research Campus Institutional Animal Care and Use Committee in the David H. Murdock Research Institute (Kannapolis, NC, USA), an AAALAC accredited animal care facility (protocol No. 12-018, approved on 10 April 2013). 

### 2.2. Body Composition and Fat/Lean Body Mass Ratios 

Animal weight and food intake (accounting for spillage) were recorded weekly for the duration of the study. Body composition analysis was performed on unanesthetized mice using EchoMRI (Echo Medical Systems, Houston, TX, USA) during the last week of the study. To determine the relative changes in skeletal muscle and adipose tissues, additional ratios of lean body mass/total body weight, fat body mass/total body weight, and fat body mass/lean body mass were calculated based on these data. 

### 2.3. Fasting Glucose and Oral Glucose Tolerance (OGTT) 

At the end of the study, mice were fasted overnight (12 h), and fasting blood glucose was determined from blood collected from a tail nick using a handheld True Metrix glucometer (Trividia Health, Fort Lauderdale, FL, USA). Immediately after, animals were challenged with an oral gavage of glucose normalized to 1.5 g/kg body weight, and blood sugar spike was monitored at 30, 60, and 120 min following the challenge. Gavage volume (μL) of 20% glucose solution was calculated for each animal as 7.5× fasted body weight (g). To identify animals with impaired glucose tolerance, area under the curve (AUC) was calculated using the linear trapezoid method and presented as arbitrary AUC units [16]. 

### 2.4. Insulin Glucose Tolerance (ITT) 

At the end of the study, but on a separate occasion, mice were fasted for 6 h, and fasting blood glucose was determined from blood collected from a tail nick using a handheld True Metrix glucometer (Trividia Health, Fort Lauderdale, FL, USA). Immediately after, animals were challenged with an intraperitoneal injection of insulin, assuming 24 IU/mg (Santa Cruz Biotechnology, Dallas, TX, USA), and normalized to 0.75 IU/kg body weight. Injection volume (μL) of 0.25 IU/mL insulin stock solution (1:400) was calculated for each animal as 3× fasted body weight (g). Disappearance of blood sugar was monitored from a tail nick at 20, 40, 80, and 120 min following the challenge. To identify animals with insulin tolerance, area under the curve (AUC) was calculated using the linear trapezoid method and presented as arbitrary AUC units [17]. 

### 2.5. RNA Extraction and cDNA Synthesis

The total RNA was isolated from tissues using TRIzol reagent (Life Technologies, Carlsbad, CA, USA) following the manufacturer’s instructions. RNA was quantified using a BioTek SynergyH1/Take 3 plate (Agilent, Santa Clara, CA, USA). The cDNAs were synthesized using 2 μg of RNA for each sample using a high-capacity cDNA Reverse Transcription kit following the manufacturer’s protocol on an ABI GeneAMP 9700 (Life Technologies).

### 2.6. RT2 Profiler qPCR Array

The resulting cDNA was amplified by real-time quantitative PCR using SYBR green PCR master mix (Life Technologies). The amplifications were performed on an ABI 7500 Fast real-time PCR using 1 cycle at 50 °C for 2 min and 1 cycle at 95 °C for 10 min, followed by 40 cycles of 15 s at 95 °C and of 1 min at 60 °C. The dissociation curve was completed with 1 cycle of 1 min at 95 °C, 30 s at 55 °C, and 30 s at 95 °C. 

Pathway expression analysis was performed initially to compare obesity-resistant NR mice to the corresponding HFD controls using a PAMM-099Z RT2 Profiler qPCR Array (Qiagen, Valencia, CA, USA) with a set of 84 genes related to a broad set of muscle functions. Following these results, a pairwise comparison among all study groups was performed using a PAMM-087Z RT2 Profiler qPCR Array (Qiagen) with a set of 84 genes to specifically focus on the mitochondrial function. 

Differentially expressed genes from the PCR array were quantified as the fold difference in the expression of each gene between the test and control samples. The scatter plot matrixes were constructed with the center line indicating a fold change ((2 ^ (−ΔCt)) of 1, and a minimum 2-fold change in gene expression was considered significant. 

### 2.7. Volatile Fecal Metabolites

Whole fecal pellets were extracted in a 10 mL sample vial containing NaCl (0.4 g), deionized water (965 µL), 5 ppm aqueous d-11 hexanoic acid (10 µL), and 3N H_2_SO_4_ (15 µL) to acidify the sample solution to pH 2. Headspace solid phase microextraction (HS-SPME) sampling was automated by a CombiPal autosampler (LEAP Technologies, Carrboro, NC, USA) by agitating the samples at 500 rpm (5 s on and 2 s off) during a 30 min equilibration at 40 °C. Volatile compounds were extracted from the headspace with a 1 cm 50/30 µm DVB/CAR/PDMS SPME fiber (Supelco, Bellefonte, PA, USA) for 30 min at 40 °C before thermal desorption into the gas chromatography inlet. Nontargeted GCxGC-ToFMS was performed on an LECO Pegasus III coupled with an Agilent GC retrofitted with a secondary oven and cryogenic modulator (Leco Corporation, St. Joseph, MI, USA). The detector voltage was optimized by ChromaTOF between −1418.0 and −1472.2 V, and masses of 25–500 were collected at a rate of 500 spectra per second, as described previously [18,19]. The abundance of each metabolite was expressed as the log2-transformed average change in the metabolite peak area from that of the HFD controls. 

### 2.8. Cell Culture

The mouse macrophage cell line RAW 264.7 (ATCC TIB-71) was maintained in DMEM (Life Technologies) supplemented with 10% fetal bovine serum, 100 IU/mL penicillin, and 100 μg/mL streptomycin (Fisher Scientific, Pittsburg, PA, USA) at a density not exceeding 5 × 10^5^ cells/mL. Passages were performed every 3–4 days in 57 cm^2^ cell-culture dishes (Nalge Nunc International, Rochester, NY, USA) maintained at 37 °C in a humidified 5% CO_2_ Thermo Forma Series II incubator (Fisher Scientific). 

### 2.9. Nitric Oxide Production and Gene Expression

For nitric oxide quantification, RAW 264.7 cells were seeded in 96-well plates in triplicate at a concentration of 5 × 10^4^ cells/well in a 200 μL culture medium and allowed to adhere for 24 h. The cells were then pre-treated with 3–300 μM dose ranges of the target metabolites and elicited with 1 μg/mL LPS for an additional 6 h. Nitric oxide released from the stimulated macrophages was quantified using a Greiss reagent system (Promega, Madison, WI, USA) and SynergyH1 microplate reader (BioTek) at 530 nm. 

For gene expression studies, RAW 264.7 cells were seeded in 24-well plates at a concentration of 5 × 10^5^ cells/well in a 1 mL culture medium and treated as described. RNA extraction, cDNA synthesis, and qPCR analysis were performed as described in Section 2.5 and 2.6, using the following set of primers: β-actin (housekeeping), forward primer: 5′-AAC CGT GAA AAG ATG ACC CAG AT-3′, reverse primer: 5′-CAC AGC CTG GAT GGC TAC GT-3′; iNOS, forward primer: 5′-CCC TCC TGA TCT TGT GTT GGA-3′, reverse primer: 5′-TCA ACC CGA GCT CCT GGA A-3′; COX-2, forward primer: 5′-TGG TGC CTG GTC TGA TGA TG-3′, reverse primer: 5′-GTG GTA ACC GCT CAG GTG TTG-3′; TNF-α, forward primer: 5′-GTT CTA TGG CCC AGA CCC TCA CA-3′, reverse primer 5′-TAC CAG GGT TTG AGC TCA GC-3′; IL-1β, forward primer: 5′-CAA CCA ACA AGT GAT ATT CTC CAT G-3′, reverse primer: 5′-GAT CCA CAC TCT CCA GCT GCA-3′; IL-6, forward primer: 5′-TAG TCC TTC CTA CCC CAA TTT CC-3′, reverse primer: 5′-TTG GTC CTT AGC CAC TCC TTC-3′; IL-17, forward primer: 5′-ATC TGG TCC TAC ACG AAG CC-3′, reverse primer: 5′-GTC CCG GAC TTC AAG ACC C-3′; IL-18, forward primer: 5′-AGG ACA AAG AAA GCC GCC TC-3′, reverse primer: 5′-TCA TTT CCT TGA AGT TGA CGC AAG AGT-3′. Fold differences in gene expression relative to the LPS-induced controls were analyzed using the ∆∆CT method and normalized with respect to the expression of β-actin. 

### 2.10. Statistical Analysis

Data were analyzed by one-way ANOVA followed by Dunnett’s multiple-range tests using Prism 8.0 (GraphPad Software, San Diego, CA, USA). All data are presented as means  ±  SEM. Significant differences were accepted when the *p*-value was <0.05. 

## 3. Results

### 3.1. Body Weight Gain and Metabolic Phenotypes

All C57BL/6J mice at the age of 4 weeks started on the respective low-fat (3.85 kcal/g) and high-fat (5.24 kcal/g) diets for 6 weeks. Due to the polygenic nature of C57BL/6J obesity, there was a significant variation in body weight gain that allowed us to select obese-prone super-responders in the upper quartile (SRs) and obese-resistant non-responders (NRs) in the lower quartile among the normal obese controls (HFD). All animals were kept on the respective diets for 8 more weeks before a series of physiological and metabolic tests were completed during week 14 of the study. A group of SR-LFD mice was further randomly selected from SR mice and fed the LFD during the second phase of the study (weeks 7–14) to evaluate the body weight loss and metabolic responses of the SR mice when switched back to the LFD [15]. 

NR mice fed the HFD showed small food-intake increases of +11.8% over HFD controls and +12.4% over SR mice that did not reach significance (*p* = 0.066). Both NR and SR mice showed increased VO_2_ oxygen consumption over the HFD controls; however, NR mice consumed +50.2% more oxygen over SR mice only during the light (sleep) cycle of the day (*p* < 0.01), thus indicating a higher resting metabolic rate (RMR). Heat production normalized to body weight was also higher in NR mice over SR mice during both light (resting, +19.5%) and dark (active, +17.3%) cycles of the day. Taken together, these data suggested that muscle tissue was a major point of physiological adaptation to the high dietary fats [15]. 

### 3.2. Relative Changes in Lean and Fat Body Mass

**Figure 1:**
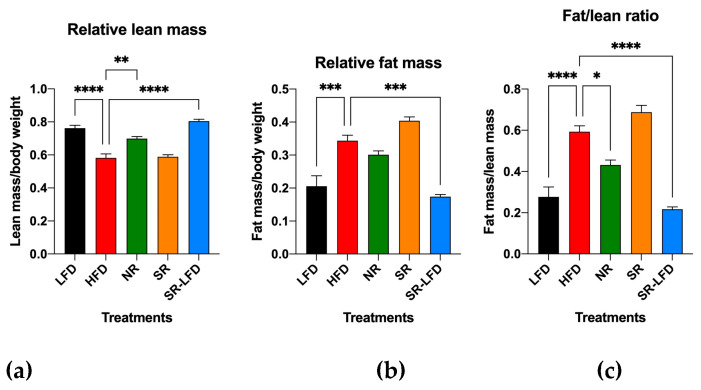
Ratios of (**a**) lean/total body mass, (**b**) fat/total body mass, and (**c**) fat/lean body mass as determined by EchoMRI body composition analysis after 14 weeks of high dietary fat challenge in lean controls (fed the LFD), obese controls (fed the HFD), obesity-resistant non-responders (NRs), obesity-prone super-responders (SRs), and super-responders fed the LFD (SRs-LFD). Results are expressed as means ± SEM (n = 8). Data were analyzed using one-way ANOVA followed by Dunnett’s multiple comparisons, * *p* < 0.05, ** *p* < 0.01, *** *p* < 0.001, **** *p* < 0.00001, versus the LFD controls.

To focus on the relative contribution of muscle tissues to the observed obesity-resistant phenotype, we quantified the relative lean mass (Figure 1a), relative fat mass (Figure 1b), and fat-to-lean-mass ratio in these animals (Figure 1c). As expected, the HFD promoted higher adiposity and therefore lower lean/total body mass ratios in HFD mice (0.58 ± 0.06) when compared to lean LFD mice (0.76 ± 0.05). Obesity-resistant NR mice showed a significant decrease in this parameter (0.69 ± 0.02, *p* = 0.002), while obesity-prone SR mice maintained lean mass levels similar to the HFD controls (0.59 ± 0.06). This suggests that SR mice failed to expand lean body mass in response to high dietary fats, and most of the body weight gain was directed towards expansion of the adipose tissue. When fat and lean body mass were compared directly (Figure 1c), this was even more obvious as the fat-to-lean mass ratio of SR mice (0.69 ± 0.07) was 60.5% higher than that of NR mice (0.43 ± 0.05, *p* < 0.0001). 

It is interesting to note that the reversal of the diet from the HFD to the LFD during the second phase of the study (8 weeks) was sufficient for SR-LFD mice to adjust their lean and fat body masses to those of the LFD controls (Figure 1c). 

### 3.3. Nutrigenomic Profiling of Muscle Tissue

**Figure 2:**
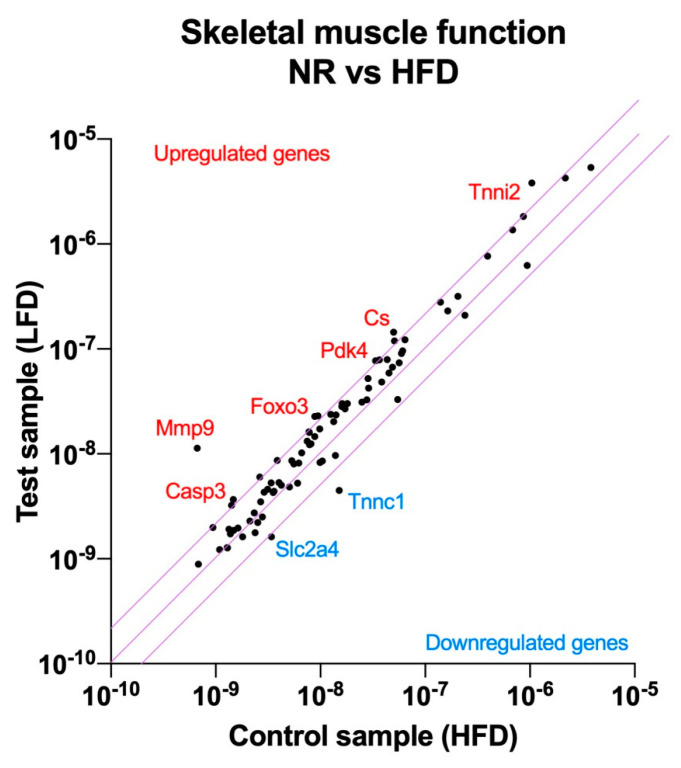
Nutrigenomic responses to high dietary fats in the obesity-resistant non-responders (NRs) versus the normal obese controls (fed the HFD). RNA was extracted from the pooled (n = 4) gastrocnemius muscle samples and RT2 Profiler Arrays were used to determine relative levels of the gene expression networks that modulate skeletal muscle physiology. The central line indicates no fold change, while the top and bottom lines indicate a 2-fold significance in the gene expression threshold.

As a first step to understand the differences between NR mice and HFD controls that allowed NR mice to remain obesity-resistant despite similar food and energy intakes, we evaluated pooled RNA samples from the gastrocnemius muscle of NR and HFD mice, as legs hold the largest muscle group that is expected to consume the most VO_2_ oxygen. The RT2 Profiler Array PAMM-099Z was used to quantify changes in the gene expression pathways related to muscle contractility, energy metabolism, slow and fast-twitch fibers, myogenesis, hypertrophy autocrine signaling, wasting, dystrophy, and insulin resistance (Figure 2).

The obese-resistant phenotype of NR mice was strongly associated with skeletal muscle tissue remodeling. Among the highest upregulated genes, matrix metallopeptidase 9 (*Mmp9*, 17.0-fold), forkhead box O3 (*Foxo3*, 2.6-fold), caspase 3 (*Casp3*, 2.5-fold), the inhibitor of kappaB kinase beta (*Ikbkb*, 2.3-fold), and interleukin (*IL1b*, 2.3-fold) indicated the upregulation of the autophagy pathway in the skeletal muscle tissue. 

Another group of upregulated genes provided further evidence about the direction of the skeletal muscle tissue remodeling. Alongside autophagy, a new signature of skeletal muscle myogenesis was evident from the expression of myocyte enhancer factor 2C (*Mef2c*, 2.3-fold), muscle receptor tyrosine kinase (*Musk*, 2.1-fold), lamin A (*Lmna*, 2.1-fold), and calpain 2 (*Capn2*, 2.0-fold). This form of myogenesis was driven in the direction of muscle fiber remodeling in favor of type 2a, fast-twitch, oxidative muscle fibers, as indicated by the higher abundance of the type 2a biomarkers: skeletal troponin I fast 2 (*Tnni2*, 3.7-fold) and myosin heavy polypeptide 2 (*Myh2*, 2.0-fold). At the same time, troponin C, the biomarker for the type 1 slow-twitch fiber, was significantly reduced (*Tnnc1*, −3.4-fold).

Finally, changes in a smaller group of genes related to nutrient partitioning and energy metabolism indicated a physiological adjustment to higher energy intakes by the upregulation of citrate synthase (*Cs*, 2.9-fold), pyruvate dehydrogenase kinase 4 (*Pdk4*, 2.3-fold), and the concurrent downregulation of the GLUT4 glucose transporter (*Slc2a4*, −2.1-fold) (Supplementary Table S1). 

### 3.4. Changes in Glucose Metabolism and Insulin Resistance

**Figure 3:**
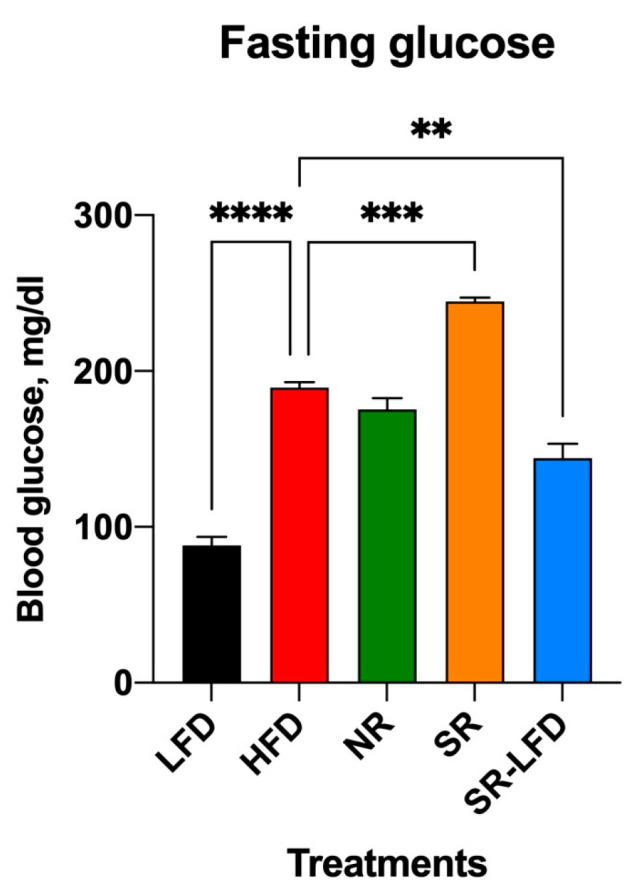
Changes in fasting blood glucose as observed after 14 weeks of high dietary fat challenge in lean controls (fed the LFD), obese controls (fed the HFD), obesity-resistant non-responders (NRs), obesity-prone super-responders (SRs), and super-responders fed the LFD (SRs-LFD). Results are expressed as means ± SEM (n = 8). Data were analyzed using one-way ANOVA followed by Dunnett’s multiple comparisons, ** *p* < 0.01, *** *p* < 0.001, **** *p* < 0.00001, versus the HFD controls.

The upregulation of citrate synthase with a simultaneous decrease in GLUT4 glucose transporter expression is highly suggestive of a metabolic adaptation in the form of blood glucose sparing to favor increased fat oxidation in the muscle tissues. To test this hypothesis, we evaluated glucose and insulin sensitivity in all study animals. Baseline blood glucose levels were measured on week 14 of the study after an overnight fast (Figure 3). As expected for diet-induced C57BL/6J obesity, the HFD controls developed high fasting blood glucose levels (189.3 ± 6.1 mg/dL) versus the lean LFD controls (88.0 ± 9.8 mg/dL, *p* < 0.0001). Resistance to obesity in NR mice was not associated with changes in fasting blood glucose levels (175.0 ± 12.8 mg/dL). SR mice also maintained high blood glucose levels (244.7 ± 4.0 mg/dL), which tended to decline in the direction of basal levels after SR-LFD mice were returned to the LFD (144.1 ± 16.1 mg/dL). 

**Figure 4:**
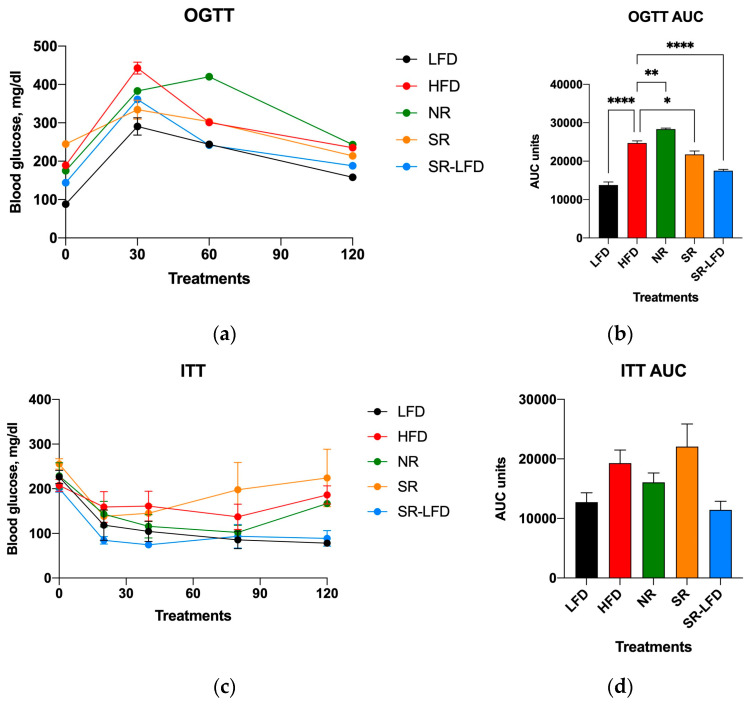
Parameters of carbohydrate metabolism affected by high dietary fats in lean controls (fed the LFD), obese controls (fed the HFD), obesity-resistant non-responders (NRs), obesity-prone super-responders (SRs), and super-responders fed the LFD at the end of the study. These include (**a**) oral glucose tolerance (OGTT) and the calculated (**b**) area under the curve (AUC), as well as an (**c**) insulin tolerance test (ITT) with the corresponding (**d**) AUC data. Results are expressed as means ± SEM (n = 8). Data were analyzed using one-way ANOVA followed by Dunnett’s multiple comparisons, * *p* < 0.05, ** *p* < 0.01, **** *p* < 0.00001, versus the HFD controls.

In line with these findings, both oral glucose tolerance (Figure 4a,b) and insulin tolerance (Figure 4c,d) were highly affected by excessive dietary fats. The OGTT data that indicated whether animals could use and store glucose normally were rather peculiar. A reduced tissue ability to remove glucose from the bloodstream was evident for all animals challenged with high dietary fats, such as the HFD, NR, and SR mice (Figure 4a,b). However, while glucose intolerance was established for these groups, it was even more pronounced in NR mice that showed peak glucose values not only at 30 min, but also at 60 min OGTT (Figure 4a). The resulting AUC for NR mice was significantly higher than that for the HFD controls (*p* = 0.004), while SR mice showed a small but significant decrease in the AUC as compared to that of HFD controls (*p* = 0.016). It is unclear what molecular mechanisms are responsible for the observed glucose sparing effect. 

The insulin tolerance determined via the ITT (Figure 4c,d) showed a matching reversal of the observed changes. SR mice tended to show increased insulin tolerance over the HFD controls, while NR mice showed a decreased tolerance, even though both trends did not reach significance (Figure 4d). It is also notable that SR-LFD mice nearly fully restored their glucose and insulin tolerance after 8 weeks on the LFD. 

### 3.5. Nutrigenomic Profiling of Skeletal Muscle Mitochondria

**Figure 5:**
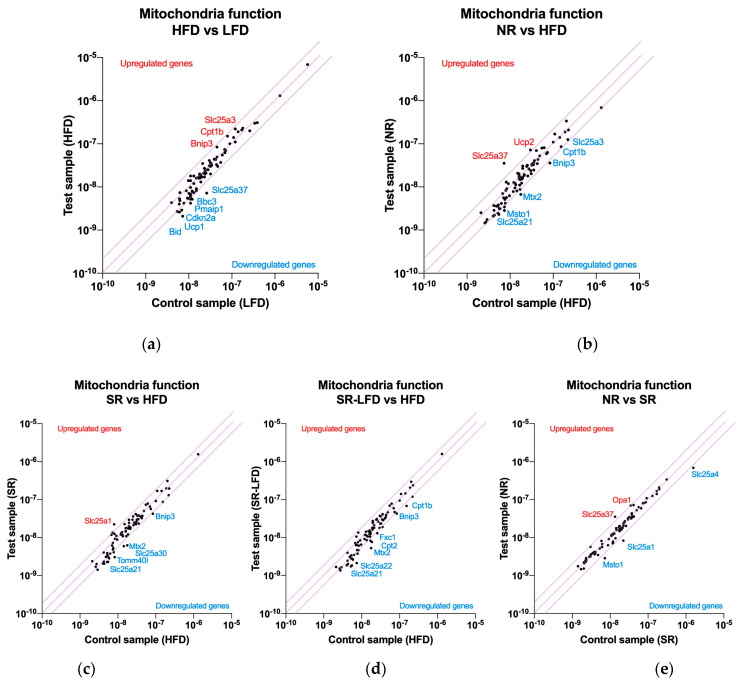
Nutrigenomic responses to high dietary fats by (**a**) lean controls (fed the LFD), (**b**) obesity-resistant non-responders (NRs), (**c**) obesity-prone super-responders (SRs), and (**d**) super-responders fed the LFD during the second phase of the study (SRs-LFD) as compared to the obese controls (fed the HFD), and (**e**) obesity-resistant non-responders (NRs) as compared to the obesity-prone super-responders (SRs). RNA was extracted from the pooled (n = 4) gastrocnemius muscle samples and the RT2 Profiler Array PAMM-087Z was used to determine relative levels of the gene expression networks that modulate muscle tissue mitochondria. The central line indicates no fold change, while the top and bottom lines indicate a 2-fold significance in the gene expression threshold.

To further understand the metabolic adaptation of muscle tissues to high dietary fats and the nutrigenomic responses associated with obesity-resistant and obesity-prone phenotypes, we evaluated pooled RNA samples from the gastrocnemius muscles of all treatment groups using the RT2 Profiler Array PAMM-087Z to target gene expression pathways related to the mitochondria. The array included networks related to mitochondrial membrane polarization, mitochondrial transport, small molecule transport, protein targeting and import to the mitochondria, inner and outer membrane translocation, mitochondrial fission and fusion, and mitochondrial localization and apoptosis (Figure 5). 

High dietary fats as a part of the HFD upregulated genes that support fatty acid oxidation, such as carnitine palmitoyltransferase Ib (*Cpt1b*) and phosphate carrier *Slc25a3* (imports of phosphate into the mitochondrial matrix), as well as mitochondrial dysfunction via BCL2/adenovirus E1B interacting protein 3 (*Bnip3*) in the near-significant 1.84- to 1.92-fold range (Figure 5a). A series of genes were downregulated, including mitoferrin 1 (*Slc25a37*), solute carriers localized in the inner mitochondrial membrane (−3.59-fold), those in the apoptosis regulatory network (*Bid*, *Pmaip1*, *Cdkn2a*, *Bbc3*, *Sfn*), and a series of solute carries in the near-significant 1.69- to 1.98-fold range. These included *Slc25a10* (an import of malate and succinate), *Slc25a13* (an import of glutamate in exchange for aspartate), *Slc25a24* (an import of ATP-Mg in exchange for phosphate), *Slc25a27* (fatty acid-activated uncoupling protein 4), and *Slc25a21* (an import of the 2-oxoadipate intermediate from the catabolism of lysine, tryptophan, and hydroxylysine). Finally, a set of three genes (*Msto1*, *Nefl*, *Uxt*) responsible for the regulation of mitochondrial distribution and morphology were also downregulated in the sub-significant −1.63- to −1.83-fold range (Supplementary Table S2). 

When compared to the HFD controls, obesity-resistant NR mice showed the capacity to significantly upregulate mitoferrin 1 (*Slc25a37*, 4.9-fold) and uncoupling protein 2 (*Ucp2*, 2.4-fold) (Figure 5b). This unique phenotype was also associated with further downregulations of the same gene expression networks as observed in the HFD controls, including solute carriers (*Slc25a10*, *Slc25a13*, *Slc25a21*, but also *Slc25a22*, *Slc25a31*, *Slc25a4*, *Slc25a2*), apoptosis (*Bnip3*, *Bbc3*), and mitochondrial distribution (*Mtx2*, *Msto1*, *Nefl*) (Supplementary Table S3). 

Obesity-prone SR mice, when contrasted against the HFD controls, showed a unique upregulation of the citrate transport protein (*Slc25a1*, 2.8-fold) responsible for the export of citrate in exchange for malate from cytosol (Figure 5c). The remaining gene expression profile was unremarkable, with similar downregulation of networks responsible for solute carriers (*Slc25a30*, *Slc25a13*, *Slc25a21*, *Slc25a31*, *Slc25a2*), apoptosis (*Bbc3*, *Bnip3*, *Cdkn2a*, *Pmaip1*), and mitochondrial distribution (*Mtx2*, *Nefl*) (Supplementary Table S4). 

SR-LFD mice fed the LFD showed a similar pattern in gene expression networks modulated by being fed the HFD (Figure 5d), including solute carriers (*Slc25a10*, *Slc25a13*, *Slc25a21*, *Slc25a22*, *Slc25a31*, *Slc25a2*, *Slc25a20*), apoptosis (*Bbc3*, *Bnip3*), and mitochondrial distribution (*Mtx2*, *Nefl*) pathways (Supplementary Table S5).

When contrasted directly to SR animals (Figure 5e), NR mice showed only a few significant differences in gene expression networks. These included a consistent upregulation of mitoferrin 1 (*Slc25a37*, 2.6-fold), optic atrophy 1 homolog protein (Opa1, 2.0-fold), translocase of outer mitochondrial membrane 40 homolog protein (Tomm40l, 1.9-fold), and uncoupling protein 2 (*Ucp2*, 1.7-fold). Only three genes were significantly downregulated, including solute carriers (*Slc25a4*, but also *Slc25a1*) and mitochondrial distribution (*Msto1*) (Supplementary Table S6). 

### 3.6. Fecal Microbiome Metabolites

The phylum-level diversity of microbiomes showed a moderate change associated with the different experimental diets. Consuming the HFD produced the typical shift in the *Firmicutes* (+15.2%) and *Bacteroidetes* (−12.9%) groups expected in the presence of high dietary fats. Obesity-prone SR mice showed a further reduction in the *Bacteroidetes* (−7.1%) group at the expense of *Actinobacteria* (+10.3%), an enterotype that persisted in SR-LFD animals as well. Obesity-resistant NR mice, on the other hand, showed an expansion of *Bacteroidetes* (+25.6%) at the expense of *Actinobacteria* (−14.5%) [15]. 

Microbiome shifts also resulted in changes in fecal metabolite signatures, including several metabolites related to short-chain fatty acids, their esters, and trans-2-dodecenol, an unsaturated fatty alcohol (Table 1). Consumption of the HFD resulted in significant increases in methyl formate, the ethyl ester of propanoic acid, 2-methyl-butanoic acid, and Z-2-dodecenol. NR and SR mice showed an opposing effect in handling volatile short-chain acid metabolites and their esters in the gastrointestinal tracts: these metabolites were lower in NR and higher in SR animals. The return of SR-LFD mice to the LFD showed a general trend to reduce these differences in the direction of the LFD metabolic signature. 

### 3.7. Inflammatory Responses

**Figure 6:**
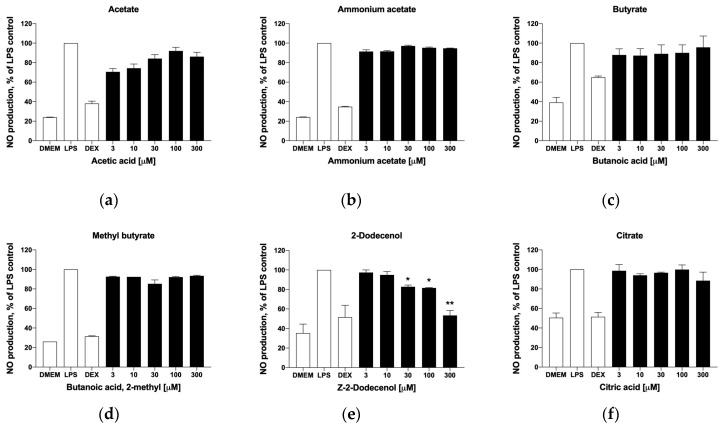
Effects of selected metabolites on nitric oxide production in activated macrophages, including (**a**) acetic acid, (**b**) ammonium acetate, (**c**) butanoic acid, (**d**) 2-methylbutanoic acid, (**e**) trans-2-dodecenol, and (**f**) citric acid. Cells were pre-treated with the target metabolites and inflammatory response was induced with 1 µg/mL LPS for 6 h. Changes in nitrite concentration as an indirect measure of nitric oxide production were reported as mean ± SEM relative to the LPS controls (* *p* < 0.05, ** *p* < 0.01).

As many of these metabolites are known to reach systemic circulation in micromolar concentrations [20], we next explored their putative effects on skeletal muscle tissues, and specifically on the inflammatory status of tissue macrophages that regulate muscle remodeling and repair. This was performed in the model LPS-stimulated mouse RAW 264.7 macrophage cell culture. In the range of physiological concentrations tested (3–300 μM), short-chain fatty acids showed no dose-dependent effects on nitric oxide production (Figure 6a–d). On the contrary, trans-2-dodecenol showed a dose-dependent suppression of nitric oxide production, thus indicating that this metabolite has a moderate anti-inflammatory effect (Figure 6e). Citrate was also tested in this model system due to the upregulation of the citrate synthase (*Cs*) and citrate transport protein (*Slc25a1*) observed in the gene expression datasets (Figure 5), but was shown to be inactive (Figure 6f). 

**Figure 7:**
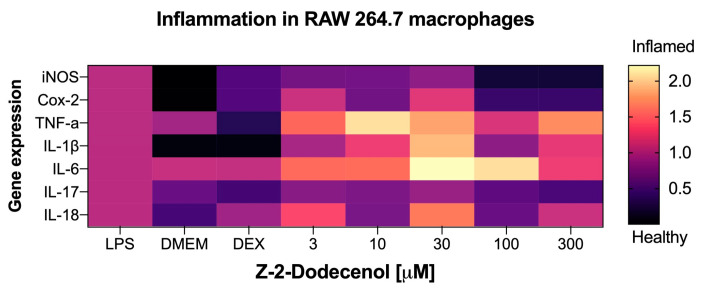
Heatmap of the anti-inflammatory effects of trans-2-dodecenol based on the qPCR gene expression profiles of key biomarkers of acute and chronic inflammation, including inducible nitric oxide synthase (iNOS), cyclooxygenase-2 (Cox-2), tumor necrosis factor alpha (TNF-α), and interleukins IL-1β, IL-6, IL-17, and IL-18. Macrophages were pre-treated with trans-2-dodecenol as specified, and inflammatory response was induced with 1 μg/mL LPS for 6 h. DMEM (baseline) and dexamethasone 10 μM (DEX) were used as negative and positive controls. Total RNAs were isolated from triplicate treatments and pooled for qPCR analysis. Fold changes in gene expression are reported as means relative to the LPS controls.

To correlate the results of the NO production assay, we next evaluated trans-2-dodecenol for its ability to modulate the expression of the pro-inflammatory gene expression network that is associated with acute and chronic inflammation. The metabolite was tested in the same dose range (3–300 μM) that was effective at reducing nitric oxide production (Figure 6e). As expected, the strongest anti-inflammatory effect associated with trans-2-dodecenol was observed in the expression levels of the inducible nitric oxide synthase (iNOS), with the expression levels returning to basal levels similar to those of non-induced controls at 100–300 μM (Figure 7). Even in a lower dose range (3–30 μM), iNOS expression was reduced by 22–34%. A similar, albeit a weaker, response was also observed for COX-2 expression, with there was 58–62% suppression of the LPS-induced levels at the higher concentration ranges of 100–300 μM. Gene expression levels for TNF-α, IL-1β, IL-6, and IL-18 were not affected, and IL-17 showed a weak response that did not reach significance. 

## 4. Discussion

Resistance to obesity is a natural phenotype that has been observed both in rodents and humans. It is described by lower body weight gain and adiposity despite the 10–25% increase in calorie intake over control obese animals fed an HFD [21]. In our previous study, we confirmed these findings by showing that obesity-resistant NR mice gained 48.6% less body weight while consuming 11.9% more calories than their obesity-prone SR counterparts, which resulted in dramatically reduced feed efficiency [15]. These data present a strong argument that obesity-resistant NR mice partition and metabolize dietary fats differently. Upon absorption, dietary fats are delivered to the gut, liver, and muscle rather than the adipose tissue within the initial 24 h after eating [11]. Primary variations in skeletal muscle tissue physiology and fat oxidation capacity may, therefore, be responsible for the initial inefficient processing of lipids that results in their excessive accumulation in the adipose tissue or the off-target ectopic deposition [22]. 

The increased ability to oxidize dietary fats in the skeletal muscle requires a set of physiological adaptations to support excessive energy fluxes. The higher proportion of lean-to-fat body mass observed in the obesity-resistant NR mice supports this conclusion, and the activity of several gene expression networks further suggests the direction in which skeletal muscle tissue adaptation takes place. In this study, we observed a simultaneous upregulation of autophagy and skeletal muscle myogenesis pathways in the NR mice, which is indicative of ongoing muscle remodeling. Healthy muscles contain a mixture of four muscle fiber types in a relative proportion according to the species and anatomical site [23]. These include type 1 (red, slow-twitch, oxidative fibers that express myosin heavy chain *Myh7*), type 2a (pink, fast-twitch, oxidative fibers that express *Myh2*), type 2x (purple, fast-twitch, glycolytic fibers that express *Myh1*), and type 2b (blue, fast-twitch, glycolytic fibers that express *Myh4*), although the latter appears to be absent in humans [24]. The existence of a continuous spectrum of hybrid fiber types, including 1/2a, 2a/2x, and 2x/2b, especially during muscle type transition and regeneration, was also observed [25]. Muscle fiber types are also tightly coupled to troponin isoforms both in rodents [26] and humans [27]. NR mice showed a very prominent gene expression signature, characterized by the upregulation of *Myh2* and troponin I2 (*Tnni2*) biomarkers, indicative of an increased presence of type 2a oxidative fibers, with a concurrent downregulation of the troponin C1 (*Tnnc1*) biomarker of type 1 fibers. The upregulation of the *Myh1* biomarker associated with type 2x fibers is also expected to increase muscle oxidative capacity, as in rodents (but not humans) type 2x fibers have moderate to strong succinate dehydrogenase activity, reflecting increased oxidative metabolism [28]. Together, these findings predict that resistance to obesity in NR mice is mediated in part by the expansion and higher oxidative function of the skeletal muscle type 2a fibers, which also explains the decreased exercise capacity observed earlier in these animals. These data were obtained from the gastrocnemius muscle, which has significantly more fast-twitch glycolytic fibers [29]. It remains to be seen whether the same shifts occur in other muscle tissues. 

The increased ability to oxidize dietary lipids was likely followed by the concurrent changes in carbohydrate metabolism. NR mice showed a diminished oral glucose tolerance (OGTT). In the adipose and muscle tissues, insulin increases GLUT4 translocation to the cell membrane, which allows for enhanced glucose uptake but these tissues [30]. Our data suggest that NR mice express GLUT4 in the muscle tissue at a level half that of the HFD controls. This adaptation may be helpful in allowing skeletal muscle to preferentially uptake and oxidize lipid substrates, although this hypothesis needs to be verified at the protein level in future studies. Further indirect support for this conclusion may come from the observed simultaneous upregulation of pyruvate dehydrogenase kinase 4 (*Pdk4*) and citrate synthase (*Cs*) transcripts. *Pdk4* is an inhibitor of pyruvate dehydrogenase that increases the influx of acetyl-CoA from β-oxidation into the TCA cycle, thereby leading to enhanced fatty acid oxidation and the slowing of glycolysis [31]. Citrate synthase, in its turn, has a key role in the TCA cycle by performing an irreversible commitment of acetyl-CoA with oxaloacetate to form citrate [32]. 

The data from the mitochondrial gene expression networks revealed the two most prominent targets associated with the obesity-resistant NR phenotype. The first target was mitoferrin 1 (*Slc25a37*), an essential iron importer for the synthesis of mitochondrial heme and iron–sulfur clusters [33]. The expression of mitoferrin 1 was suppressed by dietary fats in HFD controls as well as obese-prone SR mice, but was upregulated above the basal levels in the NR phenotype. Previously, anemia has been associated with increased fat accumulation [34], and mild iron deficiency has adversely affected lipid metabolism in rats [35]. Iron inadequacy is one of the four most common deficiencies around the world due to a variety of dietary, agricultural, and gastrointestinal factors [36]. As the mitochondria are major hubs of iron utilization and accumulation [37], it is very plausible that iron inadequacy, directly (low iron) or indirectly (inability to effectively transport iron into the mitochondria), may contribute to the development of obese phenotypes. 

The second prominent characteristic of the obesity-resistant NR phenotype in our study was the upregulation of uncoupling protein 2 (*Ucp2*). *Ucp2* dissipates the mitochondrial membrane potential as heat, thus protecting the mitochondria from reactive oxygen species overload and taking part in many adaptive metabolic processes [38], and its functionality to transport protons similarly to the archetypal protein *Ucp1*, which is activated by fatty acids and inhibited by purine nucleotides, was described previously [39]. In the latter role, Ucp2 acts as a metabolic switch that suppresses glucose utilization in the skeletal muscle in favor of utilizing lipids as major energy substrates [40]. It also catalyzes the exchange of oxaloacetate, malate, and aspartate for phosphate, and therefore regulates pyruvate oxidation in the mitochondrial matrix [41]. Another transcript revealed by the direct comparison of the obesity-resistant NR to obesity-prone SR phenotypes was optic atrophy 1 (*Opa1*), a protein essential for mitochondrial fusion, lipid, metabolism, and for supporting cellular energetics [42]. *Opa1* deficiency reduced mitochondrial respiratory capacity in white adipocytes, impaired lipolytic signaling, and promoted adipose tissue senescence [43]. In the skeletal muscles, *Opa1* fuses the inner membranes of adjacent mitochondria, allowing for an increase in oxidative phosphorylation [44], in line with the findings reported in this study. These findings need to be further validated at the protein level. 

We also observed several transcriptional changes associated with the expression of other members of the SLC25 mitochondrial carrier family [45]. The HFD was associated with the downregulation of *Slc25a10* (an import of malate and succinate), *Slc25a13* (an import of glutamate in exchange for aspartate), *Slc25a21* (an import of 2-oxoadipate), *Slc25a24* (an import of ATP-Mg in exchange for phosphate), and *Slc25a27* (fatty acid-activated uncoupling protein 4). Obesity-prone SR mice showed further downregulation of *Slc25a13*, *Slc25a21*, as well as *Slc25a2* (a transporter of the positively charged amino acids ornithine, lysine, and arginine), *Slc25a4* and *Slc25a31* (which enable ATP/ADP antiporter uncoupling activity), *Slc25a22* (an import of glutamate), and the unique upregulation of *Slc25a1* (citrate export in exchange for malate). None of these changes were counteracted in the obesity-prone NR mice, suggesting that the import of various energy substrates into the mitochondria may not be a primary mode of resistance to obesity in these animals. 

Finally, we attempted to evaluate changes in the microbiome profiles and associated alterations in short-chain acid metabolism in volatile fecal metabolomes (Table 1). The obesity-resistant NR phenotype showed a remarkable expansion of *Bacteroidetes* at the expense of a near-loss of *Actinobacteria*. As a result, the *Firmicutes* to *Bacteroidetes* ratio increased with the HFD (6.4:1) and returned to the control LFD baseline (2.29:1) in NR mice (1.63:1) [15]. This also translated to differences in fecal volatile short-chain fatty acid (SCFA) profiles that included metabolites with formate (C1), acetate (C2), propionate (C3), butyrate (C4), and isobutyrate (C5) carbon chains, as seen previously [46]. Although the majority of SCFAs are produced by the fermentation of complex carbohydrates by *Bifidobacteria* (early stages of life) and *Firmicutes* (later on), they also can be produced from the metabolism of proteins and amino acids [47]. Across all classes, the obesity-resistant NR phenotype was associated with reduced SCFA production, at least at the level of fecal SCFAs, as circulating SCFAs were not evaluated in this study. The HFD was particularly associated with the increased accumulation of methyl formate and ethyl propionate, which have the potential to exacerbate gastrointestinal and metabolic health outcomes [48,49,50]. Both metabolites were further increased in the feces of the obesity-prone SR mice, but remained at near-basal levels in the obesity-resistant NR mice, suggesting that they can be used as volatile biomarkers in studies targeting lipid metabolism and obesity. 

SCFAs are generally regarded as protective [51], but different SCFAs can have contrasting effects on lipid metabolism depending on the metabolic health of the individual [52], and often enough, increased fecal SCFAs correlate with obesity [53,54] and obesity-related dysbiosis [55]. When we tested some of these SCFA metabolites in the cell culture model of LPS-induced inflammation, they showed no defined anti-inflammatory effects, similar to what we observed in a previous study [56]. Another volatile fecal metabolite, (Z)-2-dodecenol, on the other hand, dose-dependently inhibited nitric oxide production in the LPS-induced macrophages, and its anti-inflammatory potential was supported by a dose-dependent suppression of iNOS and Cox-2 expression from the panel of genes associated with acute and chronic inflammation. (Z)-2-dodecenol was likely of dietary origin, as this metabolite can be found in soybean oil [57], a standard component of the HFDs used in this study. 

## 5. Conclusions

The findings from this exploratory study on individual obesity resistance in C57BL/6J mice reveal a complex interplay of physiological adaptations that contribute to their phenotype. The distinct metabolic traits observed in the skeletal muscle, in particular the upregulation of biomarkers for the oxidative muscle type 2a fibers, enhanced the expression of mitochondrial iron importer mitoferrin 1, and proton transport carrier *Ucp2* may suggest possible adaptive mechanisms that promote effective lipid utilization and mitigate the associated oxidative stress. Concurrently, the shifts in gut microbiota composition and fecal volatile metabolite profiles may indicate methyl formate and ethyl propionate as potential volatile biomarkers to monitor the pathophysiology and progression of obese states. 

Understanding the intricate mechanisms governing resistance to obesity in the obesity-resistant NR mice not only provides valuable insights into metabolic adaptations but also unveils potential molecular targets aimed at modulating energy metabolism and mitigating obesity-related complications. Further exploration, especially at the protein level, to validate these hypotheses and clinical studies investigating the translatability of these findings to human contexts will be crucial for harnessing the full therapeutic potential of these observations. 

## Figures and Tables

**Table 1:** Fecal volatile metabolite signatures exhibiting significant differences (* *p* < 0.0023). Abundances for each treatment group were calculated as the log2-transformed metabolite peak averages and are expressed as fold changes from those of the HFD mice.

Metabolite	LFD	HFD	NR	SR	SR-LFD
Methyl formate *	0.18	1	0.02	5.67	1.99
Heptyl formate	0.85	1	2.09	1.24	11.56
Acetic acid *	0.80	1	0.12	1.14	0.88
Ammonium acetate *	0.92	1	0.01	3.19	0.05
Ethyl propionate *	0.06	1	0.18	13.56	10.25
Propionic acid	1.21	1	0.96	1.45	0.71
Isobutyric acid	0.94	1	0.84	1.10	0.58
2,2-dimethylpropanoic acid	0.99	1	1.02	1.06	0.84
Butyric acid *	0.23	1	0.23	0.32	0.23
2-methylbutanoic acid *	0.01	1	0.17	0.01	0.01
Z-2-dodecenol *	0.01	1	0.78	0.37	0.55

## Data Availability

Data are contained within the article.

## Supplementary Materials

The following supporting information can be downloaded at: https://www.mdpi.com/article/10.3390/metabo14010069/s1, Table S1: Fold-changes in skeletal muscle function gene expression (NRs versus HFD controls); Table S2: Fold-changes in mitochondrial function gene expression (HFD versus LFD controls); Table S3: Fold-changes in mitochondrial function gene expression (NRs versus HFD controls); Table S4: Fold-changes in mitochondrial function gene expression (SRs versus HFD controls); Table S5: Fold-changes in mitochondrial function gene expression (SRs-LFD versus HFD controls); Table S6: Fold-changes in mitochondrial function gene expression (NR versus SR mice).
