# Supplementary material for: Biomarkers of Metabolic Adaptation to High Dietary Fats in a Mouse Model of Obesity Resistance
Source: Metabolites. 2024 Jan 20;14(1):69. doi: 10.3390/metabo14010069 (PMC10819356; doi:10.3390/metabo14010069)
Supplement: Supplementary file 1 [file metabolites-14-00069-s001.zip › metabolites-2800050-supplementary.pdf]

metabolites

MDPI

Article

Biomarkers of Metabolic Adaptation to High Dietary Fats in a Mouse Model of Obesity Resistance

Fadia Milhem [1,2,3], Leah M. Hamilton [2,4], Emily Skates [1], Mickey Wilson [1], Suzanne D. Johanningsmeier [5] and Slavko Komarnytsky [1,2,*]

[1] Plants for Human Health Institute, North Carolina State University, 600 Laureate Way, Kannapolis, NC 28081, USA; fjmilhem@ncsu.edu (F.M.); eskates@ncsu.edu (E.S.); mlwilso8@ncsu.edu (M.W.)
[2] Department of Food, Bioprocessing and Nutrition Sciences, North Carolina State University, 400 Dan Allen Drive, Raleigh, NC 27695, USA; lhamilton@vsu.edu
[3] Department of Nutrition, University of Petra, 317 Airport Road, Amman 11196, Jordan
[4] College of Agriculture, Virginia State University, 1 Hayden Drive, Petersburg, VA 23806, USA
[5] United States Department of Agriculture-Agricultural Research Service, Southeast Area, Food Science and Market Quality & Handling Research Unit, North Carolina State University, 322 Schaub Hall, Box 7624, Raleigh, NC 27695, USA; suzanne.johanningsmeier@usda.gov
* Correspondence: komarnytsky@ncsu.edu; Tel.: +1-(704)-250-5459

Supplementary Data

Supplementary Table S1. Fold-changes in skeletal muscle function gene expression (NRs versus HFD controls).

Fold	Gene	Gene description
17.0	*Mmp9*	Matrix metallopeptidase 9
3.7	*Tnni2*	Troponin I, skeletal, fast 2
2.9	*Cs*	Citrate synthase
2.6	*Foxo3*	Forkhead box O3
2.5	*Casp3*	Caspase 3
2.4	*Dmd*	Dystrophin, muscular dystrophy
2.3	*Mef2c*	Myocyte enhancer factor 2C
2.3	*Pdk4*	Pyruvate dehydrogenase kinase, isoenzyme 4
2.3	*Ikbkb*	Inhibitor of kappaB kinase beta
2.3	*Il1b*	Interleukin 1 beta
2.2	*Tgfb1*	Transforming growth factor, beta 1
2.2	*Ppp3ca*	Protein phosphatase 3, catalytic subunit, alpha isoform
2.1	*Musk*	Muscle, skeletal, receptor tyrosine kinase
2.1	*Actn3*	Actinin alpha 3
2.1	*Lmna*	Lamin A
2.0	*Capn2*	Calpain 2
2.0	*Myh2*	Myosin, heavy polypeptide 2, skeletal muscle, adult
2.0	*Myh1*	Myosin, heavy polypeptide 1, skeletal muscle, adult
-2.1	*Slc2a4*	Solute carrier family 2 (facilitated glucose transporter), member 4
-3.4	*Tnnc1*	Troponin C, cardiac/slow skeletal

Supplementary Table S2. Fold-changes in mitochondrial function gene expression (HFD versus LFD controls).

Fold	Gene	Gene description
1.92	Cpt1b	Carnitine palmitoyltransferase 1b, muscle
1.85	Bnip3	BCL2/adenovirus E1B interacting protein 3
1.84	Slc25a3	Solute carrier family 25 (phosphate carrier), member 3
-1.69	Slc25a21	Solute carrier family 25 (oxodicarboxylate carrier), member 21
-1.73	Immp2l	IMP2 inner mitochondrial membrane peptidase-like (S. cerevisiae)
-1.80	Timm22	Translocase of inner mitochondrial membrane 22 homolog (yeast)
-1.83	Msto1	Misato homolog 1 (Drosophila)
-1.84	Slc25a27	Solute carrier family 25, member 27
-1.84	Slc25a24	Solute carrier family 25 (phosphate carrier), member 24
-1.88	Slc25a13	Solute carrier family 25 (adenine nucleotide translocator), member 13
-1.94	Nefl	Neurofilament, light polypeptide
-1.98	Slc25a10	Solute carrier family 25 (dicarboxylate transporter), member 10
-2.09	Sfn	Stratifin
-2.31	Bbc3	BCL2 binding component 3
-2.32	Ucp1	Uncoupling protein 1 (mitochondrial, proton carrier)
-2.35	Cdkn2a	Cyclin-dependent kinase inhibitor 2A
-2.58	Pmaip1	Phorbol-12-myristate-13-acetate-induced protein 1
-3.42	Bid	BH3 interacting domain death agonist
-3.59	Slc25a37	Solute carrier family 25, member 37

Supplementary Table S3. Fold-changes in mitochondrial function gene expression (NRs versus HFD controls).

Fold	Gene	Gene description
4.9	Slc25a37	Solute carrier family 25, member 37
2.4	Ucp2	Uncoupling protein 2 (mitochondrial, proton carrier)
-1.8	Ucp1	Uncoupling protein 1 (mitochondrial, proton carrier)
-1.8	Slc25a13	Solute carrier family 25 (adenine nucleotide translocator), member 13
-1.8	Cpt1b	Carnitine palmitoyltransferase 1b, muscle
-1.8	Slc25a10	Solute carrier family 25 (dicarboxylate transporter), member 10
-1.9	Slc25a2	Solute carrier family 25 (ornithine transporter), member 2
-1.9	Nefl	Neurofilament, light polypeptide
-1.9	Slc25a4	Solute carrier family 25 (adenine nucleotide translocator), member 4
-1.9	Slc25a31	Solute carrier family 25 (adenine nucleotide translocator), member 31
-1.9	Fxc1	Fractured callus expressed transcript 1
-2.0	Timm9	Translocase of inner mitochondrial membrane 9 homolog (yeast)
-2.1	Bbc3	BCL2 binding component 3
-2.2	Slc25a22	Solute carrier family 25 (mitochondrial carrier, glutamate), member 22
-2.4	Bnip3	BCL2/adenovirus E1B interacting protein 3
-2.4	Slc25a21	Solute carrier family 25 (mitochondrial oxodicarboxylate carrier), member 21
-2.6	Msto1	Misato homolog 1 (Drosophila)
-2.6	Mtx2	Metaxin 2

Supplementary Table S4. Fold-changes in mitochondrial function gene expression (SRs versus HFD controls).

Fold	Gene	Gene description
2.8	Slc25a1	Solute carrier family 25 (citrate transporter), member 1
1.9	Slc25a37	Solute carrier family 25, member 37
-1.8	Cpt1b	Carnitine palmitoyltransferase 1b, muscle
-1.8	Pmaip1	Phorbol-12-myristate-13-acetate-induced protein 1
-1.8	Slc25a13	Solute carrier family 25 (adenine nucleotide translocator), member 13
-1.9	Slc25a2	Solute carrier family 25 (ornithine transporter), member 2
-2.0	Slc25a31	Solute carrier family 25 (adenine nucleotide translocator), member 31
-2.0	Bnip3	BCL2/adenovirus E1B interacting protein 3
-2.1	Cdkn2a	Cyclin-dependent kinase inhibitor 2A
-2.1	Nefl	Neurofilament, light polypeptide
-2.1	Timm9	Translocase of inner mitochondrial membrane 9 homolog (yeast)
-2.3	Bbc3	BCL2 binding component 3
-2.4	Slc25a21	Solute carrier family 25 (mitochondrial oxodicarboxylate carrier), member 21
-2.4	Slc25a30	Solute carrier family 25, member 30
-2.7	Tomm40l	Translocase of outer mitochondrial membrane 40 homolog-like (yeast)
-2.8	Mtx2	Metaxin 2

Supplementary Table S5. Fold-changes in mitochondrial function gene expression (SRs-LFD versus HFD controls).

Fold	Gene	Gene description
1.7	Timm22	Translocase of inner mitochondrial membrane 22 homolog (yeast)
-2.0	Bnip3	BCL2/adenovirus E1B interacting protein 3
-2.0	Slc25a20	Solute carrier family 25 (carnitine/acylcarnitine translocase), member 20
-2.0	Slc25a10	Solute carrier family 25 (dicarboxylate transporter), member 10
-2.0	Slc25a13	Solute carrier family 25 (adenine nucleotide translocator), member 13
-2.1	Fxc1	Fractured callus expressed transcript 1
-2.1	Slc25a2	Solute carrier family 25 (ornithine transporter), member 2
-2.2	Timm9	Translocase of inner mitochondrial membrane 9 homolog (yeast)
-2.2	Slc25a31	Solute carrier family 25 (adenine nucleotide translocator), member 31
-2.3	Cpt1b	Carnitine palmitoyltransferase 1b, muscle
-2.3	Nefl	Neurofilament, light polypeptide
-2.4	Cpt2	Carnitine palmitoyltransferase 2
-3.0	Bbc3	BCL2 binding component 3
-3.0	Slc25a21	Solute carrier family 25 (mitochondrial oxodicarboxylate carrier), member 21
-3.4	Mtx2	Metaxin 2
-3.5	Slc25a22	Solute carrier family 25 (mitochondrial carrier, glutamate), member 22

Supplementary Table S6. Fold-changes in mitochondrial function gene expression (NR versus SR mice).

Fold	Gene	Gene description
2.6	Slc25a37	Solute carrier family 25, member 37
2.0	Opa1	Optic atrophy 1 homolog (human)
1.9	Tomm40l	Translocase of outer mitochondrial membrane 40 homolog-like (yeast)
1.7	Ucp2	Uncoupling protein 2 (mitochondrial, proton carrier)
-2.3	Slc25a4	Solute carrier family 25 (adenine nucleotide translocator), member 4
-2.5	Msto1	Misato homolog 1 (Drosophila)
-2.7	Slc25a1	Solute carrier family 25 (citrate transporter), member 1
